# Tolerance and Adherence of Patients with Chronic Chagas Disease Treated with Benznidazole

**DOI:** 10.1590/0037-8682-0384-2022

**Published:** 2023-01-23

**Authors:** Cristina Vázquez, Elisa García-Vázquez, Bartolomé Carrilero, Marina Simón, Fuensanta Franco, María Asunción Iborra, Luis Javier Gil-Gallardo, Manuel Segovia

**Affiliations:** 1 Hospital Clínico Universitario Virgen de la Arrixaca, Microbiology Service, El Palmar, Murcia, Spain.; 2 Universidad de Murcia, Microbiology and Genetics Department, Murcia, Spain.

**Keywords:** Chagas, Benznidazole, Trypanosoma cruzi, Tolerance, Adherence

## Abstract

**Background:**

Chagas disease (CD) treatment is commonly associated with a high incidence of adverse effects. It is crucial to study and update these adverse effects to improve the existing knowledge of which drugs to use and to clarify the information presented to patients.

**Methods:**

We analyzed the adverse effects of benznidazole in two cohorts of patients: a large retrospective study and a small prospective study.

**Results:**

This large retrospective study described the most and least common adverse effects in our area and characterized our Chagas disease population. This prospective study, along with a close follow-up of the treatment, detected more adverse effects and enhanced the patients’ perception of the disease and treatment.

**Conclusions:**

This information is important for preventing non-medical-related withdrawals and for removing baseless fears. Better knowledge of patients could help us provide better care.

## INTRODUCTION

Chagas disease affects over six million people worldwide and is characterized by a short acute phase and a long chronic phase. Although treatment efficacy is well established in acute cases (60-76%)^1^ ^-^ ^3^, the role of trypanocide therapy in the chronic phase has not been clearly recognized until several observational studies and a few small, randomized trials have pointed toward the convenience of treating patients with chronic Chagas disease to slow the progression of the illness^4^ ^-^ ^6^. In addition, a major trial in 2006 concluded that treatment was associated with a reduced risk of cardiomyopathy progression in patients in the undetermined phase of the disease and did not have serious adverse effects^7^. On this basis, the World Health Organization (WHO) recommends offering treatment to patients during the early chronic phase, especially in adults with no symptoms, although in some cases, the potential benefits of medication to prevent or delay the development of Chagas disease should be weighed against the duration of treatment and the risk of adverse reactions^8^. In 1973, benznidazole was one of the two drugs approved for Chagas disease treatment and was the most prescribed trypanocide in our area. The associated adverse effects include clinical events such as abdominal pain, hypersensitive skin reactions, weight loss, headache, nausea, vomiting, urticaria, itching, anorexia, diarrhea, and, less frequently, laboratory events, such as neutropenia and eosinophilia^9^. Previous studies have examined the safety of Chagas disease treatment in small cohorts^10^ ^-^ ^13^. The present study retrospectively evaluated the safety profile of benznidazole over 11 years in our experience at the Tropical Medicine Unit of a third-level hospital in a non-endemic area in southeast Spain and adds a weekly follow-up of 43 chronic Chagas patients from their diagnosis until the end of their trypanocide treatment. The objective of our study is to add to the existing knowledge on the safety profiles of specific treatments.

## METHODS

### Retrospective study

From 2007 to 2017, all adult Chagas patients diagnosed and treated at our Tropical Medicine Outpatient Clinic were registered in the database. Our standard protocol covered demographic data (sex, age, weight, and country and region of origin) as well as treatment data (drug prescription, treatment adherence, adverse effects, and possible second treatments and their characteristics). Patients under 18 years of age, pregnant women, a small cohort treated with nifurtimox during a supply interruption, patients who did not return for follow-up, and those who did not receive treatment were excluded. 

Chagas disease was diagnosed using two complementary methods: a chemiluminescent microparticle immunoassay (ARCHITECT Chagas®, Abbott) and an indirect immunofluorescence assay (Inmunofluor Chagas kit, Biocientifica S.A.). Owing to its higher availability in our area, benznidazole was administered to all patients as the first choice treatment^14^. The prescribed dosage was 5-7.5 mg/kg divided into three daily doses for 60 days^15^. The cumulative dose did not exceed 18 g of benznidazole to prevent adverse effects such as polyneuritis and bone marrow depression^16^. 

Adverse effects were defined based on the Common Terminology Criteria for Adverse Events version 4.0^17^. Adverse effects that could not be included in any category were considered as “Others”.

Logistic regression models (univariate and multivariate) were used to determine the possible association between demographics and adverse effects.

Before treatment initiation, 38 patients were randomly tested for benznidazole allergies to prevent benznidazole-associated skin disorders. A patch test was performed to confirm this diagnosis. The procedure consisted of attaching an occlusive patch containing benznidazole dispersed in petrolatum to the patient’s upper back. After a 48-hour exposure, an initial reading was taken, followed by a second reading at 72 h and a third reading at 92 h. The results were interpreted according to the European Society of Contact Dermatitis guidelines^18^.

### Prospective study

Between July and November 2017, all newly diagnosed patients were offered weekly follow-up of their Chagas disease treatment. First, a questionnaire was used to determine whether the patient had already suffered from any of the symptoms described as adverse effects of trypanocide (Appendix 1). During treatment, weekly phone calls were made to assess adherence and to ask about possible adverse effects (Appendix 2). Patients who were under 18 years of age, pregnant women, patients who did not take the medication, and those who did not answer at least the last control call were excluded. The Naranjo algorithm was used to assess the causality of adverse drug reactions^19^. During active follow-up, laboratory testing was not performed unless clinically necessary.

## RESULTS

### Retrospective study

Between 2007 and 2017, 1581 adult patients were diagnosed in our Tropical Medicine Unit, of which 726 met the inclusion criteria and two were excluded due to lack of information in their clinical records about adverse effects associated with medication; therefore, 724 patients were included in this phase. The most frequent country of origin was Bolivia (97.9%), followed by Ecuador, Argentina, Brazil, and Paraguay, with fewer than 10 individuals from each country. The demographic data and adverse effects are listed in Table 1. In the studied cohort, 15.3% of patients were unable to complete treatment, either because of adverse effects or other reasons, mostly personal, such as forgetfulness and returning to their country. In this retrospective study, no information was available concerning the time of onset or the severity of all adverse events (except skin disorders). Among the patients who did not complete their treatment due to adverse effects (68.8%), the most frequent causes were moderate (31 patients), mild (25), and severe (12) dermatological reactions. 

**TABLE 1:** Demographic and adverse effects of the retrospective cohort.

	N (%)	Mean (SD)
**Sex**		
Man	236 (32.6)	
Woman	488 (67.4)	
**Age**		38.7 (9.4)
**Benznidazole brand**		
ABARAX	418 (57.7)	
ROCHAGAN	306 (42.3)	
**Adverse effects**		
No	396 (54.7)	
Yes	328 (45.3)	
**Treatment fulfillment**		
No	111 (15.3)	
Yes	613 (84.7)	
**Gastrointestinal disorders**		
No	684 (94.5)	
Yes	40 (5.5)	
1 adverse effect	38 (5.2)	
2+ adverse effects	2 (0.3)	
**Neurologic disorders**		
No	649 (89.6)	
Yes	75 (10.4)	
1 adverse effect	68 (9.4)	
2+ adverse effects	7 (1)	
**Other disorders**		
No	670 (92.5)	
Yes	54 (7.5)	
1 adverse effect	47 (6.5)	
2+ adverse effects	7 (1)	
**Skin disorders**		
No	461 (63.7)	
Mild	161 (22.2)	
Moderate/Severe	102 (14.1)	

**SD:** standard deviation.

Out of 38 patients tested with the benznidazole patch test, four were positive and 34 were negative. Among the negative patients, 20 developed adverse effects during treatment, 18 of whom developed skin disorders classified by severity as six mild, 10 moderate, and two severe.

Statistical analysis showed that sex and the presence of skin disorders was relevant in the univariate analysis (Table 2). Women were 1.7 times more likely to abandon treatment than men (OR = 1.70, 95% CI = 1.07-2.63), while patients with mild or moderate/severe skin disorders were 1.88 and 8.09 times more likely to withdraw (OR = 1.88, 95% CI = 1.10-3.21; OR = 8.09, 95% CI = 4.88-13.41).

**TABLE 2:** Univariate and multivariate logistic regression to identify risk factors for treatment withdrawal.

	Treatment fulfillment			Univariate			Multivariate	
	No	Yes		OR (CI 95%)	***p*-value**		OR (CI 95%)	***p*-value**
**Sex**								
Man	26 (11)	210 (89)						
Woman	85 (17.4)	403 (82.6)		1.70 (1.07-2.73)	0.026		1.37 (0.83-2.25)	0.219
**Age**	38.6 (9.4)	38.7 (9.4)		1.00 (0.98-1.02)	0.911		1.00 (0.98-1.03)	0.899
**Benznidazole brand**								
ABARAX	64 (15.3)	354 (84.7)						
ROCHAGAN	47 (15.4)	259 (84.6)		1.00 (0.67-1.51)	0.986		1.32 (0.84-2.09)	0.232
**Gastrointestinal disorders**								
No	107 (15.6)	577 (84.4)						
Yes	4 (10)	36 (90)		0.60 (0.21-1.72)	0.341		0.60 (0.20-1.84)	0.375
**Neurologic disorders**								
No	99 (15.3)	550 (84.7)						
Yes	12 (16)	63 (84)		1.06 (0.55-2.03)	0.865		0.89 (0.43-1.83)	0.753
**Other disorders**								
No	97 (14.5)	573 (85.5)						
Yes	14 (25.9)	40 (74.1)		2.07 (1.08-3.94)	0.027		1.27 (0.61-2.63)	0.526
**Skin disorders**								
No	41 (8.9)	420 (91.1)						
Mild	25 (15.5)	136 (84.5)		1.88 (1.10-3.21)	0.02		1.84 (1.07-3.16)	0.028
Moderate/Severe	45 (44.1)	57 (55.9)		8.09 (4.88-13.41)	<0.001		7.80 (4.58-13.28)	<0.001

**OR:** odds ratio. **CI:** confidence interval

Only skin disorders showed relevance in multivariate analysis, and people with mild or moderate/severe skin disorders were1.84 and 7.8 times more likely to discontinue treatment, respectively (OR = 1.84, 95% CI = 1.07-3.16 and OR = 7.80, 95% CI = 4.58-13.28).

### Prospective study

Between July and November 2017, 60 patients recently diagnosed with Chagas disease were offered a weekly follow-up during their treatment, but only 43 people fulfilled the inclusion criteria. Sixteen patients were excluded because of concurrent personal situations that prevented them from taking the medication (such as non-Chagas-related hospital admission) or adherence to follow-up (such as returning to their country). All of them were from Bolivia except for one patient who was from Ecuador; 58% were women, and the median age was 41.5 years old (range:21-67). All patients experienced at least one adverse effect, accounting for 156 episodes of adverse effects (Table 3). Most patients (23 [53.5%]) reported them within the first week (range,1-8 weeks; median duration: six days). Twenty-seven (62.8%) patients had at least one dermatological adverse effect, 29 (67.4%) had gastrointestinal symptoms, 24 (55.8%) had neurologic symptoms, and 25 (58.1%) had miscellaneous symptoms. In terms of severity, 116/156 (74.4%) adverse effects were mild, 38/156 (24.4%) were moderate, and 2/156 (1.3%) were severe; all were resolved without sequelae. The treatment withdrawal rate was 20.9%, and the most frequent cause was moderate dermatological reaction (8/9). During the study, all adverse reaction episodes (156) were recorded and classified using the Naranjo algorithm to assign causality^19^. The majority (120/156 [76.9%]) of the reactions were classified as “possible” adverse effects, 35/156 (22.4%) were classified as “probable,” and only 1/156 (0.6%) were classified as “doubtful”. 

**TABLE 3:** Adverse effects of benznidazole and symptom severity and duration.

	Frequency (AEs, % of Patients Reporting)		Severity, n (%)				Length in time (days), median (IR)
			Mild	Moderate	Severe		
Adenopathies	3 (7.0)		3 (100)				2 (1-7)
Anorexia	2 (4.7)		2 (100)				37 (14-60)
Apathy	2 (4.7)		2 (100)				15 (15-15)
Burning feet	1 (2.3)			1 (100)			7 (7-7)
Constipation	1 (2.3)		1 (100)				4 (4-4)
Cough	1 (2.3)			1 (100)			4 (4-4)
Diarrhea	2 (4.7)		2 (100)				7 (7-7)
Dyspnea	1 (2.3)		1 (100)				7 (7-7)
Drowsiness	4 (9.3)		4 (100)				5 (2.5-10.5)
Fever	4 (9.3)		1 (25)	2 (50)	1 (25)		3 (1.5-4)
Flatulence	2 (4.7)		1 (50)	1 (50)			11 (7-15)
Flu syndrome	1 (2.3)			1 (100)			30 (30-30)
Gastrointestinal pain	11 (25.6)		10 (90.9)	1 (9.1)			30 (7-30)
General discomfort	2 (4.7)		2 (100)				7 (7-7)
Generalized edema	5 (11.6)		2 (40)	3 (60)			5 (5-7)
Headache	17 (39.5)		12 (70.6)	5 (29.4)			7 (3-50)
Increased appetite	3 (7.0)		3 (100)				15 (7-40)
Increased blood pressure	1 (2.3)		1 (100)				7 (7-7)
Insomnia	4 (9.3)		3 (75)	1 (25)			40 (30-55)
Itchy tongue	1 (2.3)		1 (100)				10 (10-10)
Laryngeal inflammation	1 (2.3)		1 (100)				4 (4-4)
Leukopenia	1 (2.3)				1 (100)		7 (7-7)
Lower limb pain	1 (2.3)		1 (100)				3 (3-3)
Nausea	15 (34.9)		13 (86.7)	2 (13.3)			7 (3-15)
Oral pain	1 (2.3)			1 (100)			3 (3-3)
Palpitations	2 (4.7)		2 (100)				11 (7-15)
Paresthesia	13 (30.2)		13 (100)				20 (15-45)
Pyrosis	6 (14.0)		2 (33.3)	4 (66.7)			20 (15-60)
Skin disorders (mild)	12 (27.9)		12 (100)				7 (4.5-12.5)
Skin disorders (moderate)	15 (34.9)			15 (100)			5 (1-7)
Vertigo	5 (11.6)		5 (100)				15 (2-15)
Vomiting	3 (7.0)		3 (100)				3 (2-15)
Weakness	9 (20.9)		9 (100)				14 (7-28)
Weight loss	4 (9.3)		4 (100)				30 (18.5-30)

**AE:** adverse effects. **IR:** interquartile range.

## DISCUSSION

The success of a treatment for Chagas disease is limited by its adverse effects, which determine whether the patient will complete the regimen. In addition, a controversial issue in the treatment of adults in the chronic stage is that tolerance to medication declines with age^11^ ^,^ ^20^. Although treatments are not classified as first- and second-line, benznidazole is usually the first choice over nifurtimox because of its supposedly milder adverse effects^14^. We evaluated the adverse effects of benznidazole in a cohort of 724 patients who had been treated in our Tropical Medicine Outpatient Clinic for 11 years. The most frequent benznidazole-related adverse effects were mild and moderate/severe dermatological reactions (~14%-22%), and the second most frequent were neurologic disorders (10%). Compared with previous retrospective studies^16^ ^,^ ^21^ ^-^ ^24^, our patients had fewer gastrointestinal disorders (only 5% versus 20%-30%). A limitation of our study was the exclusion of patients who did not return for follow-up, which might introduce bias, as patients with more severe adverse effects may have decided not to return to the clinic, although this might also mean that they simply had not taken the medication.

Although retrospective studies may be associated with a lack of quality in data collection, our study included a prospective cohort of 43 newly diagnosed Chagas disease patients who fulfilled the inclusion criteria. In this prospective cohort, we recorded a high incidence of adverse effects (100% of the patients), and gastrointestinal problems prevailed as a cluster; however, headache was the most commonly reported adverse effect overall, closely followed by nausea and moderate dermatological effects. There was also a higher incidence of neurological symptoms (24 [55,8%]) compared with previous studies^10^ ^-^ ^12^, perhaps due to the classification of headache as a general symptom in other studies and a neurological symptom in this study^17^. To assign causality, the Naranjo algorithm was used^19^, and it was concluded that more than 75% of the recorded adverse effects could be considered “possible”. This algorithm was not used in this retrospective study because of the lack of detailed health reports required before and during treatment. The treatment withdrawal rate was 20.9%, which is slightly higher than that in other prospective studies^7^ ^,^ ^11^ ^,^ ^12^ ^,^ ^25^, although one study reported a higher rate (29%)^10^. The withdrawals in our cohort were due to various reasons, such as adverse reactions or personal (non-medical) decisions. Patients’ cultural backgrounds and insufficient knowledge about their illness can cause them to underestimate the disease, be afraid of possible adverse effects of treatment, or both^26^ ^,^ ^27^.

An allergy screening test (patch test) prior to benznidazole treatment was introduced to enhance therapy tolerance, either by changing the first-line treatment or by prescribing corticosteroids to mitigate the adverse dermatological effects. Nonetheless, the low sensitivity of this test (53%) may prevent it from predicting the occurrence or severity of skin disorders.

Differences in the adverse effect rates between our prospective and retrospective cohorts may be explained by a more detailed recording of health events through closer follow-up, as stated in previous studies^20^ ^,^ ^28^.

Conclusions cannot be drawn from a small study; however, a close follow-up revealed more adverse effects, both common and new, than retrospective registries. On the other hand, there is no apparent correlation between withdrawals and close follow-up; in fact, the prospective study illustrated more withdrawals than the retrospective study. However, the sole cause of these withdrawals in the prospective study was the adverse effects. Having a person in continued contact with the patient prevents withdrawals due to forgetfulness, underestimation of the disease, or apprehension. In addition, the possibility of recording adverse effects, even if the patient is going to return to their home country before the physician’s appointment, is an advantage.

Treatment of patients with chronic Chagas disease with medication is not free of adverse effects. Although several studies have demonstrated the utility of benznidazole in diminishing illness progression, its associated adverse effects remain a matter of concern. A closer follow-up may prevent unnecessary treatment withdrawal and allow early detection of adverse effects. Given that this level of supervision is often impossible based on our close patient interactions during the prospective study, we propose the following recommendations:

Inform the patient about the disease in a way that he/she can understand, and actively question the patient about thoughts and doubts. Thus, we can prevent non-medical withdrawals.

Inform the patient about the most frequent and severe adverse effects in an educational way to help him/her better identify the adverse effects.

Do not underestimate minor but frequent adverse effects such as headache, nausea, or abdominal pain. 

More attention should be paid to neurological effects (paresthesia or headache) when questioning the patient about possible adverse events during treatment.

Have an experienced team available to evaluate and tackle adverse effects to help improve completion rates.

## Figures and Tables

**APPENDIX 1:**
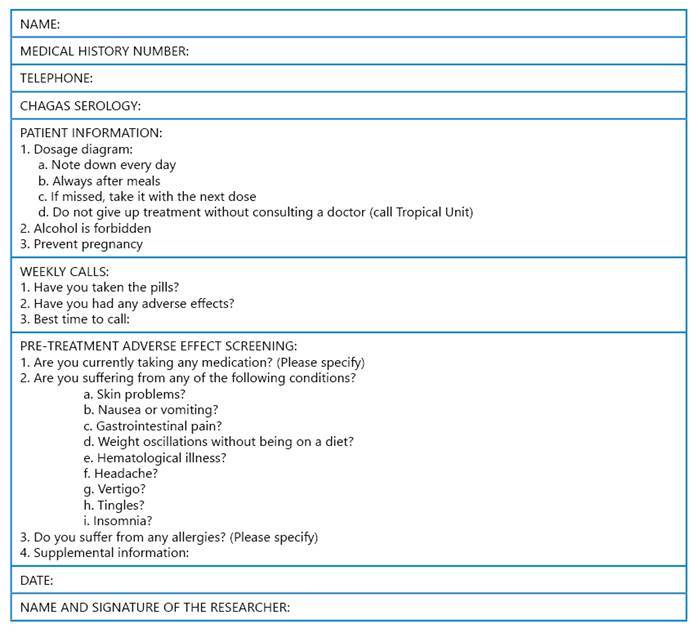
Pre-treatment questionnaire.

**APPENDIX 2:**
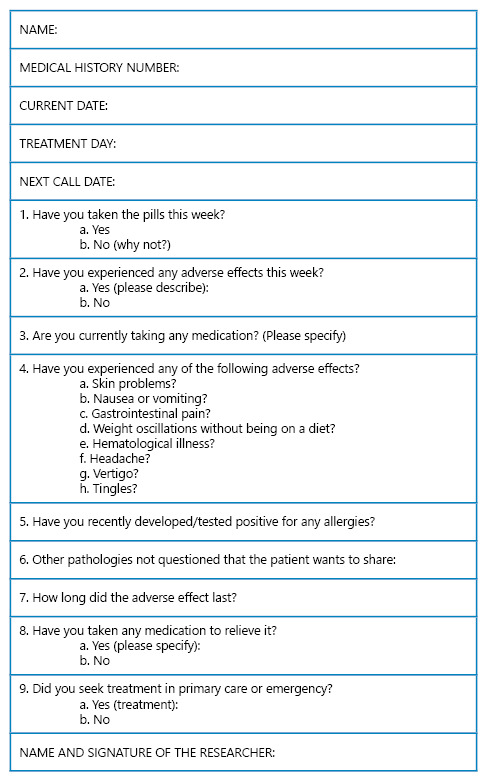
Weekly phone call questionnaire template.
